# Toxicology of tramadol following chronic exposure based on metabolomics of the cerebrum in mice

**DOI:** 10.1038/s41598-020-67974-8

**Published:** 2020-07-07

**Authors:** Wei Xia, Guojie Liu, Ziyi Shao, Enyu Xu, Huiya Yuan, Junting Liu, Lina Gao

**Affiliations:** 1 School of Forensic Medicine, China Medical University, No. 77 Puhe Road, Shenbei New District, Shenyang, 110014 China; 2 School of Fundamental Sciences, China Medical University, Shenyang, 110014 China

**Keywords:** Biochemistry, Molecular biology

## Abstract

Tramadol is an opioid used as an analgesic for treating moderate or severe pain. The long-term use of tramadol can induce several adverse effects. The toxicological mechanism of tramadol abuse is unclear. Metabolomics is a very useful method for investigating the toxicology of drug abuse. We investigated the impact of chronic tramadol administration on the cerebrum of mice, focusing on the metabolites after tramadol administration. The mice received 20 or 50 mg/kg body weight tramadol dissolved in physiological saline daily for 5 weeks via oral gavage. Compared with the control group, the low dose tramadol group showed seven potential biomarkers, including gamma-hydroxybutyric acid, succinate semialdehyde, and methylmalonic acid, which were either up- or down-regulated. Compared with the control group, the high dose tramadol group showed ten potential biomarkers, including gamma-hydroxybutyric acid, glutamine, and O-phosphorylethanolamine, which were either up- or down-regulated. The up-regulated gamma-hydroxybutyric acid and the down-regulated succinate semialdehyde revealed that the neurotransmitter system was disrupted after tramadol abuse. Compared with the low dose tramadol group, there were twenty-nine potential biomarkers in the high dose tramadol group, mainly related to the pentose phosphate pathway and glycerophospholipid metabolism. In conclusion, metabolomics in the tramadol abuse group demonstrated that long-term tramadol abuse can result in oxidative damage, inflammation, and disruption of the GABA neurotransmitter system, which will help to elucidate the toxicology of tramadol abuse.

## Introduction

Tramadol is an effective analgesic agent for the treatment of moderately severe pain^1^. Tramadol is considered to exert analgesic effects by binding the μ-opioid receptors and modulating the noradrenergic, GABAergic and serotonergic systems^2, 3^, or by acting as a serotonin-norepinephrine (NE) reuptake inhibitor^4^. Tramadol in clinical not only can be used in general surgery, obstetrics, pediatrics and the treatment of oral surgery, as well as a variety of acute postoperative pain, is also used to relieve chronic pain, such as cancer^5^. Because its analgesic action time is longer;its analgesia intensity decreased slowly, it is a relatively ideal drug for chronic pain medication.Most common side effects of tramadol include nausea, vomiting, sweating, fatigue, sedation^6, 7^, and dry mouth^8^. More severe side effects include angioedema, increased effect of anticoagulants, hypoglycemia^7, 9^ and serotonin toxicity^8^.Tramadol was identified as a controlled substance in the USA and UK (schedule IV drug) in 2014^10, 11^, and is also a controlled psychotropic substance in China, as more young people are abusing it to obtain psychological satisfaction. Having a lower affinity for the μ-opioid receptor, Tramadol has shown to have a lower risk for addiction with chronic use when compared with other opiates e.g. morphine and oxycodone^9, 12^. Thus, many studies on the risks of opioid abuse have excluded tramadol^12–14^. Mohamed HM^4^ reported that chronic exposure to tramadol induces oxidative damage, inflammation, and apoptosis. However, little research on long-term tramadol abuse^4^ has been reported. Moreover, the toxicity mechanism of long-term exposure to tramadol is unclear.

Metabolome refers to the collection of all small molecular weight metabolites of an organism or cell in a specific physiological period, and generally refers to small molecular metabolites with a relative molecular weight less than about 1,000 Da^15^. The purpose of metabolomics is to investigate metabolite changes in biological systems (cells, tissues, etc.) after stimulation or disturbance^16^. By screening the different metabolites between the experimental group and the control group, the biological processes associated with differential metabolites can be studied to reveal the mechanism of life activities involved. Metabolomics is an omics that is closer to phenotype, which can reflect the physiological state of an organism more directly and accurately. Recently, metabolomics has been used to investigate the mechanisms involved in poisoning or drug abuse^17^.

As more young people are abusing tramadol to obtain the “high”, it is essential to research the tramadol’s effects on cerebrum. In this study, we observed the changes of metabolites in the cerebrum of mice with chronic exposure to tramdol to investigate the toxicology and the potential adverse reactions of tramadol using the metabolomics.

## Materials and methods

### Chemicals and reagents

HPLC-grade methanol was obtained from the CNW Technology Company (Beijing, China). Pyridine and methylhydroxylamine hydrochloride were purchased from Adamas Industrial, Inc. (Shanghai, China). l-2-Chlorphenylalanine (purity > 98%) was obtained from Hengbai Biotechnology Company (Shanghai, China). BSTFA (with 1% TMCS, v/v) was purchased from Regis Technology (Shanghai, China). The BCA protein assay kit and total superoxide dismutase (SOD) activity detection kit were obtained from Beyotime Biological Reagent Co., Ltd. (Shanghai, China). The malondialdehyde (MDA) detection kits were purchased from BestBio Biological Reagent Co., Ltd. (Shanghai, China). GC–MS testing was conducted by Novogena Co.,Ltd. (Beijing, China).

### Instrumentation and conditions

According to the methods published by Gao et al. ^18^, we set the parameters as following: Agilent 7,890 gas chromatography (Agilent Technologies, USA) was coupled with a Pegasus HT time-of-flight mass spectrometer (LECO Corporation, USA). The DB-5MS column (30 m × 0.25 mm, 0.25 μm film thickness) was from J&W Scientific (Folsom, CA, USA). A 1 μL aliquot of the analyte was injected in splitless mode. Helium was used as the carrier gas, the front inlet purge flow was 3 mL/min, and the gas flow rate through the column was 1 mL/ min. The initial temperature was maintained at 50℃ for 1 min, then raised to 310℃ at a rate of 20℃/min, then maintained at 310℃ for 6 min. The injection, transfer line, and ion source temperatures were 280℃, 280℃, and 250℃, respectively. The energy in the electron impact mode was 70 eV. The mass spectrometry data were acquired in full-scan mode with the m/z range of 50–500 at a rate of 12.5 spectra per second after a solvent delay of 4.8 min.

### Animal treatment and sample collection

Kunming mice (originated from Swiss mice, Changsheng Biotechnology Co., Ltd. Shenyang, China) with a body weight of 35 ± 5 g were used in this study. Eighteen male mice were randomly divided into three groups, including the control group (n = 6), the low dose tramadol group (n = 6) and the high dose tramadol group (n = 6). The mice were fed according to the breeding regulations and adapted to the environment for 1 week. All mice were housed at the Laboratory Animal Research Center of China Medical University at a temperature of 22℃ and a natural light–dark cycle. All experimental procedures were conducted according to the Institutional Animal Care guidelines and were approved as ethical by the Administration Committee of Experimental Animals at the Laboratory Animal Center of China Medical University No. 20181027.

### Experimental design

The animals were randomly allocated into three groups (n = 6) as follows:

- Group I (control): Mice received physiological saline via oral gavage daily for 5 weeks.
- Group II (20 mg tramadol): mice received 20 mg/kg/day tramadol dissolved in physiological saline via oral gavage for 5 weeks.
- Group III (50 mg tramadol): mice received 50 mg/kg/day tramadol dissolved in physiological saline via oral gavage for 5 weeks.

The dosage of tramadol was adjusted according to changes in body weight. At the end of the experiment, the mice were euthanized via compressed gas in their home cage by trained personnel. Death was confirmed after checking for lack of respiration and faded eye color in each mouse. The brain was excised and the cerebrum quickly separated. Parts of the cerebral cortex were stored at − 80 °C for detection of SOD activity and MDA content. Parts of the cerebral cortex were homogenized in cold phosphate-buffered saline (PBS), centrifuged, and the clear homogenate was collected for biochemical assays.

Mice were administered tramadol, which induced seizure-like activity. The frequencies of specific observation criteria of seizure-like activity were recorded in a composite manner based on observations spanning the first 3 h after administration on the thrity-fifth day after tramadol administration. The frequencies were summed to obtain the most representative seizure severity score. The criteria comprised facial movements, head nodding, forelimb clonus, jerky movements, falling and clonic seizures^19^.

### Metabolite extraction

Sample preparation for untargeted metabolomic profiling was carried out as described by Gao et al. ^18^. Take50 ± 1 mg cerebrum sample into the 2 ml EP tubes, extracted with 450µL extraction liquid (V methanol:V chloroform = 3:1), add 10 µl of L-2-chlorophenylalanine (1 mg/ml stock in dH2O) as internal standard, vortex mixing for 30 s; homogenized in ball mill for 4 min at 45 Hz, then ultrasound treated for 5 min (incubated in ice water); centrifuge for 15 min at 12000 rpm 4℃; transfer the supernatant 200 µL into a fresh 1.5 ml EP tubes. The supernatant was completely dried in a vacuum concentrator without heating; 60 μL methoxyamine hydrochloride (20 mg/mL in pyridine) was added and then incubated for 30 min at 80℃. Next, 80 μL of BSTFA reagent (1% TMCS, v/v) was added to the sample aliquots, and incubated for 1.5 h at 70℃. All samples were analyzed by gas chromatography coupled to a Pegasus HT time-of-flight mass spectrometer (GC-TOF–MS).

### Quantification of SOD and MDA in cerebrum homogenates

Levels of SOD and MDA in cerebrum homogenates were evaluated using detection kits, according to the manufacturer’s instructions.

### Data analysis

Chroma TOF 4.3X software (LECO Corporation) and the LECO-Fiehn Rtx5 database were used for raw peaks exacting, data baseline filtering and calibration of the baseline, peak alignment, deconvolution analysis, peak identification, and integration of the peak area^20^. Both the mass spectrum match and retention index match were considered for the identification of the metabolites. Partial least squares (PLS) analysis was performed at SIMCA-P (version 13.0, Umetrics, Sweden)^21, 22^. Different variables correlating with tramadol toxicity were monitored as follows: first, the VIP value should be greater than 1.0. Second, to eliminate the probability of false positives, an adjusted P value from the nonparametric Mann–Whitney U test (PASW Statistics 19, SPSS Inc., Chicago, IL, United States) should be lower than 0.05^23^. Third, the value of the area under the receiver operating characteristic (AUC-ROC) curve was calculated in PASW Statistics 19 (SPSS Inc., Chicago, IL, United States), and the variables were discarded when AUC-ROC ≤ 0.75. Moreover, the classification performance was considered excellent when AUC-ROC > 0.9 ^24^. Metabolite heat maps were produced in MultiExperiment View (Version 4.9.0). The changes in metabolites in each group were shown by a volcano map. The Kyoto Encyclopedia of Genes and Genomes (KEGG) pathway database^25–27^ was used to perform enrichment pathway analysis of differentially changed metabolites.

The levels of SOD and MDA were present as mean ± standard deviation (SD) and analyzed by SPSS 26.0 (SPSS Inc., Chicago, USA). The one-way ANOVA testing was used and *p* < 0.05 was considered statistically significant.

### Statement on the welfare of animals

All procedures performed in studies involving animals were in accordance with the ethical standards of the institution or practice at which the studies were conducted (the Administration Committee of Experimental Animals at the Laboratory Animal Center of China Medical University. 20181027).

## Results

### Effect of tramadol hydrochloride on seizure severity score in mice

**Figure 1:**
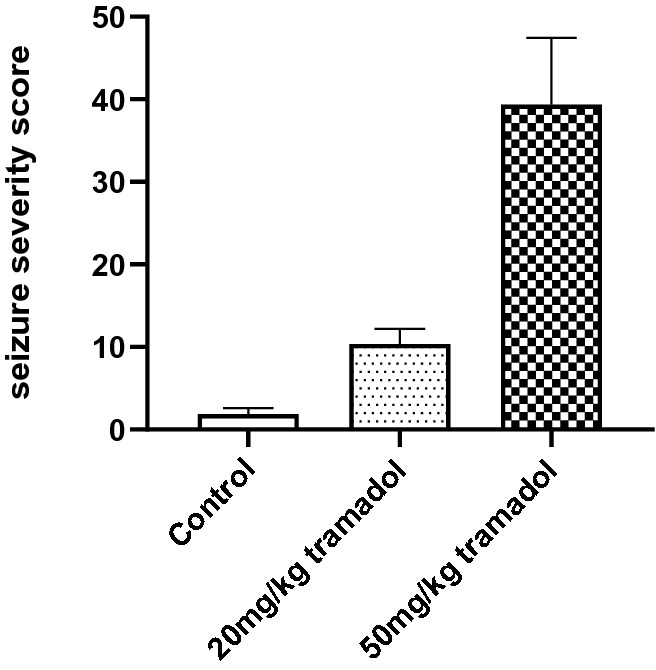
Tramadol-induced seizures in mice were assessed in terms of the composite seizure severity score. Values are expressed as mean ± S.E.M. Statistical analysis of the results was performed using one-way ANOVA. There are significant difference (*P* ˂ 0.05) between any two groups.

Tramadol administration was observed to cause the production of seizure-like behavioral syndrome in mice assessed by the frequency of facial movements, head nodding, forelimb clonus, falling and clonic seizures. In addition, the severity caused by tramadol was dose-dependent (Fig. 1).

### Metabolomics results

Representative GC–MS results obtained from the cerebrum of mice are shown in supplement Fig. 1. Detailed information, including peak assignment and multiplicity for the analyzed metabolites are listed in Tables 1, 2, and 3.

**Table 1:** The distinguished different metabolites between the low dose group and the control group.

No	Name	Pvalue	ROC	VIP	Trend
1	Succinate semialdehyde	0.00538	0.916667	1.150771	Down
2	Lactic acid	0.008525	1	1.210481	Down
3	Phenylacetaldehyde	0.003297	0.944444	1.273551	Down
4	Methylmalonic acid	0.0052	0.916667	1.049064	Down
5	Alpha-Aminoadipic acid	2.83E-06	1	1.428335	Up
6	Monoolein	0.016790	0.888888	1.840082	Down
7	γ-Hydroxybutyric acid	0.029341	0.805556	4.068085	Up

**Table 2:** The distinguished different metabolites between the high dose group and the control group.

No	Name	Pvalue	ROC	VIP	Trend
1	Phosphatidyl ethanolamine	0.010121	0.916667	1.481372	Down
2	Fructose	0.046633	0.916667	1.423181	Down
3	Glutamine	0.04178	0.66666	2.10707	Down
4	Oxalic acid	0.001415	0.972222	1.084615	Up
5	Alpha-Aminoadipic acid	0.007458	0.833333	1.31662	Up
6	Myo-inositol	0.012556	0.888889	1.48524	Up
7	γ-Hydroxybutyric acid	0.032065	0.752222	4.38976	Up
8	Fructose 2,6-biphosphate	0.04663	0.91666	1.42318	Down
9	Fructose-6-phosphate	0.01255	0.88888	1.48524	Down
10	6-Phosphate gluconic acid	0.03075	0.83333	1.44251	Down

**Table 3:** The distinguished different metabolites between the low and high dosage tramadol groups.

No.	Name_des	Pvalue	ROC	VIP	Up. Down
1	Glutamine	0.01263	0.916667	1.61973	Down
2	Phosphatidyl ethanolamine	0.001618	1	1.519291	Down
3	Fructose	0.030563	0.833333	1.211801	Down
4	Dehydroascorbic Acid	0.012467	1	1.282237	Down
5	Glucose	0.015118	0.944444	1.031273	Down
6	Galactose	0.01019	0.944444	1.263042	Down
7	Lysine	0.015042	0.888889	1.166203	Down
8	Mannitol	0.000155	0.972222	1.893367	Down
9	N-alpha-Acetyl-L-ornithine	0.014213	0.888889	1.254009	Down
10	Lipoic acid	0.012495	0.888889	1.235809	Down
11	Pantothenic acid	0.002624	0.944444	1.539796	Down
12	Gluconic acid	0.002237	0.972222	1.232332	Down
13	Fructose 2,6-biphosphate	0.000463	0.972222	1.722629	Down
14	5-Hydroxyindole-acetic acid	0.005185	0.944444	1.341267	Down
15	Oleic acid	0.000283	1	2.473685	Down
16	Stearic acid	0.004576	0.944444	1.370856	Down
17	Spermidine	0.005586	0.916667	1.269467	Down
18	Fructose-6-phosphate	0.001791	1	2.264901	Down
19	6-Phosphate gluconic acid	0.017809	0.888889	1.170693	Down
20	Pelargonic acid	0.000378	1	1.08774	Down
21	Thymine	0.034584	0.833333	1.092957	Down
22	Oxalic acid	5.98E-05	1	1.503521	Up
23	Azelaic acid	0.040329	0.833333	1.63793	Up
24	Sucrose	7.84E-05	1	3.291901	Up
25	Prostaglandin A2	0.005454	0.944444	1.511029	Up
26	Caprylic acid	0.037437	0.916667	1.059006	Up
27	Capric Acid	0.002148	1	1.303616	Up
28	γ-Hydroxybutyric acid	0.00391	1	1.01887	Up
29	N-Acetyl-L-leucine	0.006074	0.972222	3.048863	Up

**Figure 2:**
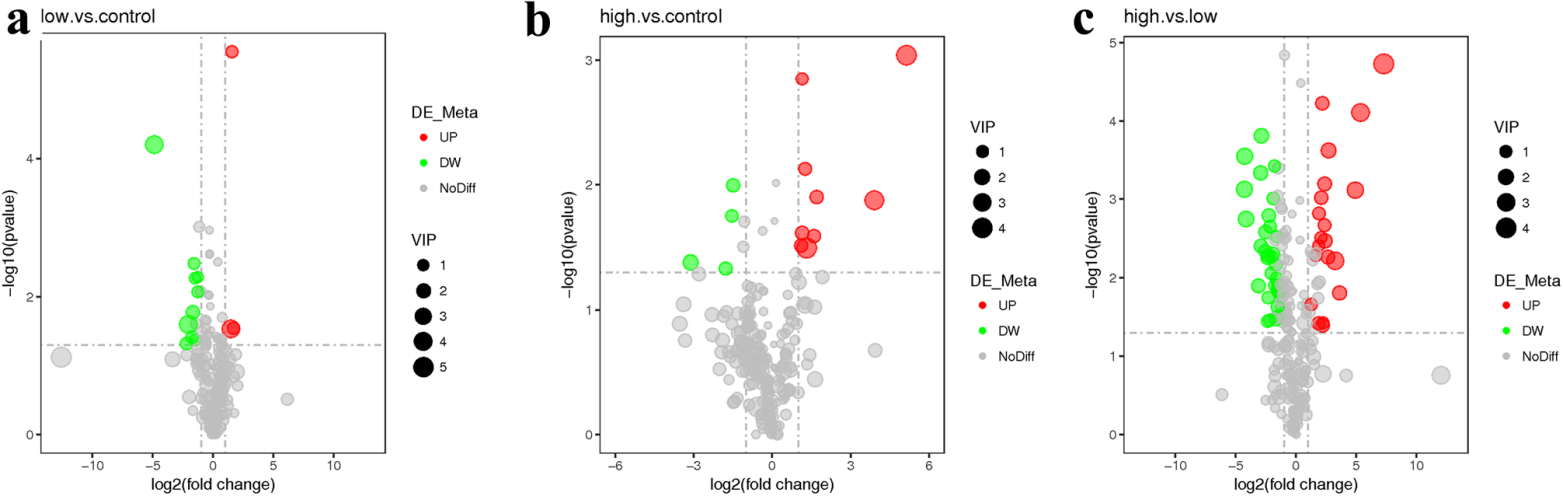
The volcano map of differentially changed metabolites between any two groups. (**a**) shows the difference in metabolites between the low dose tramadol group and the control group; (**b**) shows the difference in metabolites between the high dose tramadol group and the control group; (**c**) shows the difference in metabolites between the high dose tramadol group and the low dose tramadol group. Red represents up-regulation, green represents down-regulation, and gray represents no distinguishable difference between any two groups. VIP represents the importance projection value of this substance obtained in the PLS-DA model.

The changes in metabolites among the groups were observed in the volcano map of differentially changed metabolites, as shown in Fig. 2. From the supplement Fig. 2, there are significant different between any two groups.

**Figure 3:**
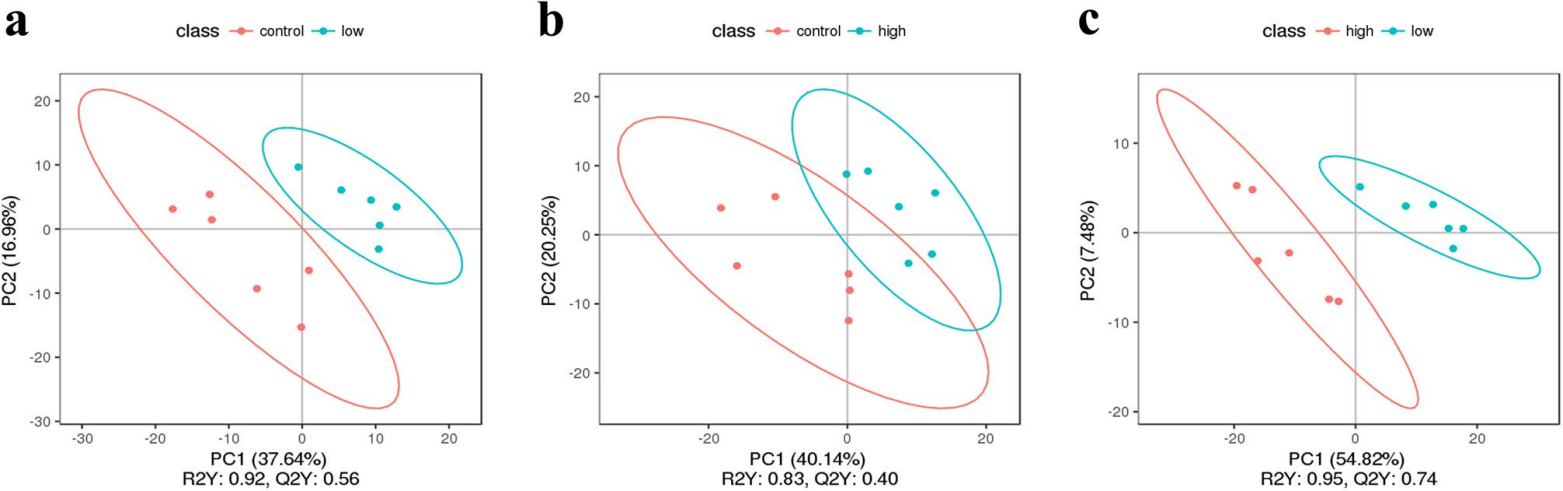
PLS-DA score. (**a)** shows the PLS-DA between the low dose tramadol group and the control group; (**b)** shows the PLS-DA between the high dose tramadol group and the control group; (**c**) shows the PLS-DA between the high dose tramadol group and the low dose tramadol group.

Partial Least Squares Discriminant Analysis (PLS-DA) is a supervised discriminant analysis statistical method. This method uses partial least square regression to establish a model of the relationship between metabolite expression and sample category to predict the sample category^28^. The PLS-DA model of each comparison group was established, and the model evaluation parameters (R2, Q2) were obtained by sevenfold cross-validation (seven cycles of interactive validation). If R2 and Q2 were close to 1, the model was more stable and reliable, as shown in Fig. 3.

**Figure 4:**
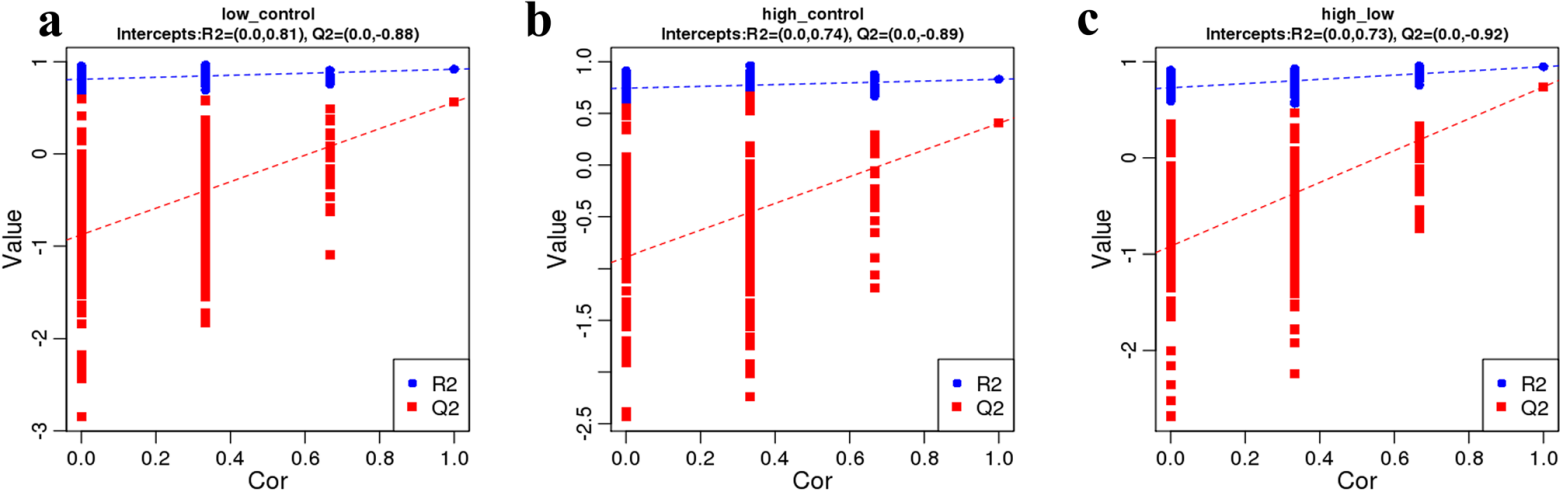
PLS-DA validity. The quality of the fitting model can be explained by the R^2^ and Q^2^ values. R^2^ represents the variance explained in the model and indicates the goodness of fit. Q^2^ represents the variance in the data predicted by the model and indicates the predictability. (**a**) shows the PLS-DA validity between the low dose tramadol group and the control group; (**b**) shows the PLS-DA validity between the high dose tramadol group and the control group; (**c**) shows the PLS-DA validity between the high dose tramadol group and the low dose tramadol group.

In order to judge the quality of the model, the model was also sorted to verify whether the model was "overfitting". The lack of "overfitting" indicates that the model can better describe the samples and can serve as the premise for finding the biomarker group of the model, while "overfitting" indicates that the model is not suitable for describing the samples and should not be used for later analysis of the data. When R2 is greater than Q2 and the intercept of the Q2 regression equation with the Y-axis is less than zero, the model is reliable, as shown in Fig. 4. Clustering, or Cluster analysis, involves grouping a set of objects in such a way that objects in the same cluster are more similar to each other than to those in other clusters to some extent. As shown in Supplementary Fig. 2, the high dose tramadol group, low dose tramadol group and the control group showed a clear separation in the clustering analysis of metabolites.

**Figure 5:**
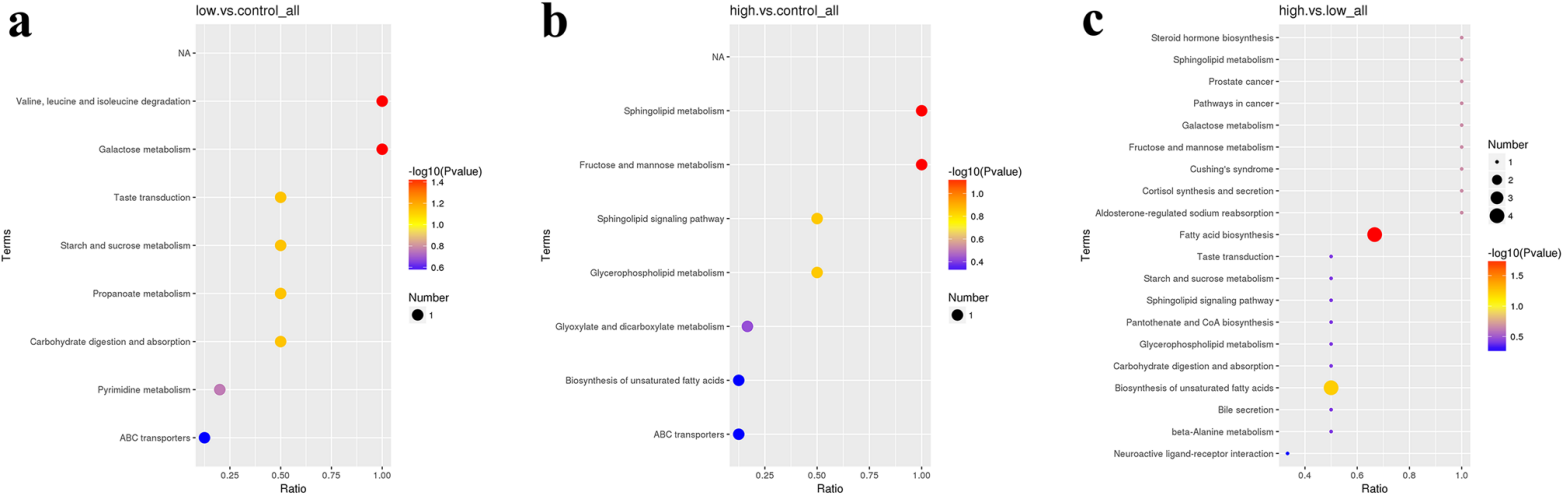
KEGG enrichment bubble chart (**a**) shows the comparison between the low dose tramadol group and the control group; (**b**) shows the comparison between the high dose tramadol group and the control group; (**c**) shows the comparison between the high dose tramadol group and the low dose tramadol group. The abscissa in Fig. 5 is the ratio of the number of differentially changed metabolites in the corresponding pathway to the total number of identified metabolites. The higher the ratio, the higher the concentration of differentially changed metabolites in the pathway. The color of the dot represents the P-value of the hypergeometric test. The smaller the *P* value, the greater the reliability and the more statistically significant the test. The size of the dot represents the number of differentially changed metabolites in the corresponding pathway. A larger point size represents a greater number of differentially changed metabolites in the pathway.

We identified differentially changed metabolites between the following group comparisons: low dose tramadol group vs. the control group (Table 1); high dose tramadol group vs. the control group (Table 2); high dose tramadol group vs. low dose tramadol group (Table 3). The differentially changed metabolites observed between the low dose tramadol group and the control group mainly participated in valine, leucine and isoleucine degradation, and galactose metabolism (Fig. 5a). As shown in Fig. 5b, these differentially changed metabolites mainly participated in sphingolipid, fructose, and mannose metabolism in the high dose tramadol group and the control group. The differentially changed metabolites observed between the high dose tramadol group and the low dose tramadol group mainly participated in fatty acid biosynthesis and the biosynthesis of unsaturated fatty acids (Fig. 5c).

### SOD activity and MDA content

**Figure 6:**
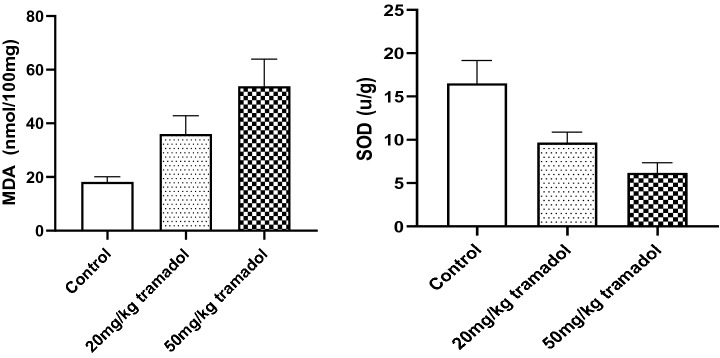
Tramadol induces lipid peroxidation, and suppresses SOD activity. MDA was increased in the cerebrum of mice receiving 20 or 50 mg/kg tramadol for 5 weeks. Data are expressed as Mean ± SEM, n = 6. The statistical analysis was performed using one-way ANOVA and *P* < 0.05 was considered statistically significant.

Oxidative stress is a key factor that leads to mitochondrial damage. Oxidative stress results in a significant increase in MDA levels as well as a decrease in SOD level. As shown in Fig. 6, compared with those in the control group, the content of MDA in the brain tissue of the high tramadol group was significantly higher, and the activity of SOD in the brain tissue was significantly lower (MDA (mmol/100 mg protein) 53.8 ± 9.22 vs. 18.2 ± 1.77; and SOD (U/g protein) 6.17 ± 1.07 vs. 16.5 ± 2.43). Compared with that in the control group, the content of MDA in the low tramadol group was significantly higher, and the activity of SOD was significantly lower (MDA (mmol/100 mg protein) 36 ± 6.25 vs. 18.2 ± 1.77; and SOD (U/g protein) 9.67 ± 1.11 vs. 16.5 ± 2.43). There were significant differences in the MDA content and SOD activity between the low tramadol group and the high tramadol group (MDA (mmol/100 mg protein) 36 ± 6.25 vs. 53.8 ± 9.22; SOD (U/g protein) 9.67 ± 1.11 vs. 6.17 ± 1.07).

## Discussion

Tramadol is a centrally acting analgesic used for the treatment of moderate-to-severe pain. Although tramadol was classified as having a low abuse potential by the U.S. Drug Enforcement Agency, the adverse reactions related to tramadol are still a concern. The present study investigated the effect of chronic tramadol administration on the cerebrum of mice based on metabolomics, with a focus on oxidative stress and inflammation as adverse reactions of tramadol.

With regard to the dosage of tramadol in animal experiments, we set the dose at 20 mg/kg and 50 mg/kg, which was consistent with a previous study^29^. Compared to the median lethal dose in mice (200–300 mg/kg), the doses used in our animal experiments were lower; however, it is better to study the long-term side effects of tramadol as a painkiller. Toxic reactions to tramadol, such as seizures and irritability, were observed in the tramadol group ^30^.

PLS-DA (Fig. 3) indicated significant differences in metabolic patterns between any two groups (the control group, the low and high dosage tramadol group). The PLS-DA graph confirmed that the model had good stability and predictability. KEGG enrichment analysis (Fig. 5) revealed differences in the metabolic patterns between any two groups. This was mainly reflected in the increase in the content of lactic acid, phenylacetaldehyde, alpha-aminoadipic acid and the decrease in methylmalonic acid and monoolein which participate in valine, leucine and isoleucine degradation, galactose metabolism, carbohydrate digestion and absorption in the low dose tramadol group. As shown in Table 1, decreased Monoolein, a kind of glycerides of unsaturated fatty acids, may be caused by oxidative damage. Aminoadipic acid (2-AAA), an intermediate metabolite of lysine metabolism, could modulate insulin secretion^31^.

In the high dosage tramadol group, this was mainly reflected in the increase in oxalic acid, alpha-aminoadipic acid, myo-inositol and the decrease in phosphatidyl ethanolamine and mannitol which participate in sphingolipid metabolism, fructose and mannose metabolism, and the sphingolipid signaling pathway. From Table 2,decreased fructose 2,6-biphosphate, fructose-6-phosphate and 6-phosphate gluconic acid, which take part in pentose phosphate pathway, indicated that the pentose phosphate pathway was activated to produce more NADPH to defense oxidation damage.Down-regulated fructose indicated that the energy metabolism was disturbed when exposed to high dosage tramadol (50 mg/kg). In the high dose tramadol group, up-regulated oxalic acid, the end product of vitamin C, that can be used as an antioxidant for scavenging oxygen radicals, represented vitamin C consumption after oxidative damage. Moreover, phosphatidyl ethanolamine was decreased during lipid peroxidation, and was consistent with the detection of MDA. In this study, the oxidative damage caused by tramadol was reflected by the determination of metabolites and MDA.Some reference^32^ reported that myo-inositol was thought to possess anti-oxidant properties, its elevated contents may highlight the importance of oxidative stress in disrupted balance of metabolic pathways.

**Figure 7:**
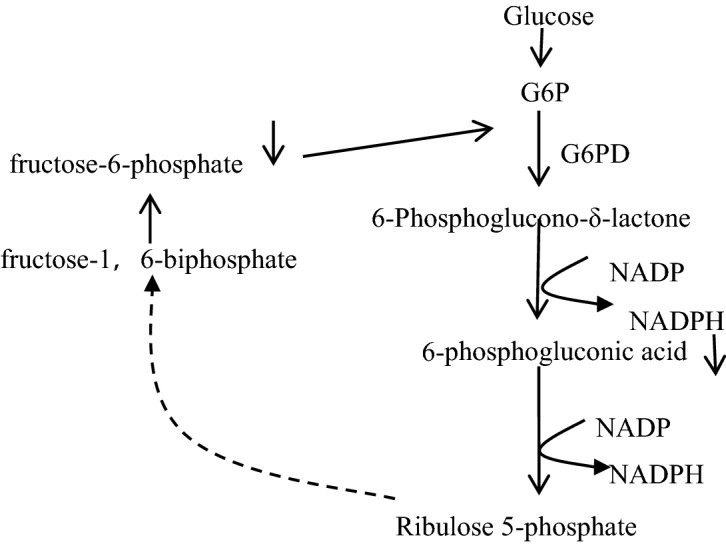
The effects of pentose phosphate pathway after tramadol treatment [fructose-6-phosphate and 6-phosphogluconic acid decreased in the tramadol group (50 mg/kg)].

From Fig. 2 and Table 3, it can be seen that there were more differentially changed metabolites between the high dose tramadol group and the low dose tramadol group, which indicated a dose-dependent effect, and was consistent with the content of MDA and SOD. Up- regulated oxalic acid and down-regulated dehydroascorbic acid indicated that oxidative damage increase with the dose increasing. Other organic acids such as caprylic acid, capric acid, pelargonic acid and azelaic acid, are also intermediates of fatty acid metabolism, were up regulated, which may be related with fatty acid oxidation disorders^33^.With increased tramadol dose, fatty acid biosynthesis and the biosynthesis of unsaturated fatty acids were disrupted, such as decreased oleic acid and stearic acid, which also reflected the oxidative damage caused by tramadol. By measuring MDA and SOD, it can be seen that there was a dose-dependent relationship, as shown in Fig. 6. Moreover, compared with the low dose tramadol group, the pentose phosphate pathway (PPP) was stimulated in the high dose tramadol group. The decrease of intracellular fructose- 2, 6-bisphosphonate inevitably leads to the slowdown of glycosysis pathway, which leads to the accumulation of fructose- 6-phosphate, which will stimulate the pentose phosphate pathway, produces ribose and NAPDH, and increases the intracellular reduced glutathione. As shown in Fig. 7, the down regulation of fructose-6-phosphate and 6-phosphate gluconic acid revealed that the PPP produced more NADPH to support antioxidant defenses^18^. As shown in Fig. 6, pretreatment with tramadol had a dose-dependent oxidative damage effect. In addition, fructose, glucose, galactose, and fructose-6-phosphate took part in galactose metabolism. These metabolites revealed that energy metabolism was affected in the high dose tramadol group. Serotonin is degraded to 5-hydroxyindole-acetic acid (5-HIAA) by monoamine oxidase. In the high dose tramadol group, the content of 5-HIAA was decreased compared with the low dose tramadol group. A unique adverse reaction of tramadol is serotonin syndrome^34^, and there is a certain relationship with the metabolics pathway of serotonin. In the high dose tramadol group, down regulated phosphatidyl ethanolamine and up regulated myo-inositol revealed the distortion of compositional phospholipids, while in the low dose tramadol group, similar metabolite changes were not found. We suspect that only exposure to high doses (over 50 mg/kg) of tramadol will influence phospholipid metabolism.

MDA is an aldehyde produced during the process of lipid peroxidation caused by free radicals. MDA can be used as a cross-linking agent to promote the cross-linking of nucleic acids, proteins and phospholipids and to change the function of biological macromolecules. The content of MDA can reflect the degree of lipid peroxidation. The role of SOD is to remove superoxide ions, the precursor of H_2_O_2_ and OH·, in order to protect cells from damage caused by toxic oxygen free radicals. Increased MDA levels and decreased SOD activity can trigger oxidative stress, leading to cell damage and even cell death, as shown in Fig. 6. The results showed that chronic tramadol administration significantly increased levels of MDA and suppressed SOD activity. Increased reactive oxygen species (ROS) can induce peroxidation of cell membrane lipids and can damage DNA and proteins, resulting in cell death. These results were consistent with the results of metabolomics.

**Figure 8:**
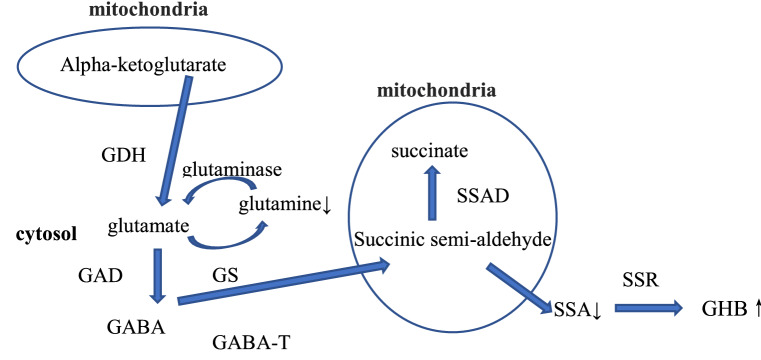
Metabolic pathway for the synthesis of gamma-hydroxybutyric acid (GHB) and glutamate/glutamine shuttle in neurons. Succinic semi-aldehyde (SSA) is formed in the mitochondria by GABA-transaminase (GABA-T). SSA can either be oxidized to succinate by succinic semialdehyde dehydrogenase (SSADH) in the mitochondria or transported to the cytosol where it is reduced to gamma-aminobutyric acid (GABA) by succinic semialdehyde reductase (SSR). Glutamate can form GABA by glutamate decarboxylase (GAD). Glutamine is broken down to glutamate under the action of glutaminase. Glutamate is converted to glutamine by glutamine synthetase (GS). In the low dose tramadol group, the content of SSA was down regulated and the content of GHB was up regulated. In the high dose tramadol group, the content of glutamine was down regulated and the content of GHB was up regulated.

It was noteworthy that the change of some neurotransmitters was observed as the dose increases, such as succinate seminaldehyde, gamma-hydroxybutyric acid and glutamine. As shown in Table 1, in the low dose tramadol group, up-regulation of gamma-hydroxybutyric acid (GHB) and down regulation of succinic semialdehyde (SSA) indicated that the GABAergic system was disrupted. Maitre et al.^35^ reported that GHB modulated GABAergic activity in some regions of the brain. The GABAergic system has been investigated extensively as a major inhibitory neurotransmitter system strongly associated with drug-dependence^36^. With the dose of tramadol abuse increasing, in the high dose tramadol group, besides upregulated GHB, glutamine was down regulated, which was suspected to be influenced by the glutamate-glutamine shuttle. In conclusion, the GABAergic system was influenced after tramadol abuse as shown in Fig. 8, which correlated with the adverse reaction of tramadol resulting in seizures. Tramadol is a non-selective opioid receptor agonist^37^. Moreover, overactivation of opioid receptors has been shown to accelerate seizure activity in various experimental animals ^38^.Therefore, it maybe possible that the stimulation of tramadol induced opioid receptor might mediate the seizurogenic effect of tramadol. In addition, seizures induced by overactivation of opioid receptor have been associated with significant inhibition of GABA receptors^19, 39^. Furthermore, Rehni et al.^40^ reported that activation of opioid receptor may result in the GABA neurotransmitter system’s inhibition, that might be responsible for the seizurogenicity of tramadol. By studying the metabolomics of tramadol abuse, we also found that the GABA neurotransmitter system was disrupted. Moreover, as shown in Fig. 1, there was a dose-dependent relationship between tramadol and seizure severity, that similar result also was found by Rehin et al.^40^. Thus, it can be concluded that there may be a correlation between inhibition of the GABA neurotransmitter system and the seizurogenicity of tramadol.

In conclusion, tramadol abuse may be a risk factor for impaired mental and emotional health, which could be mediated via the GABA neurotransmitter system. Moreover, long-term abuse of tramadol could cause oxidative damage and lipid peroxidation. These results were also found in the low and high dose tramadol groups based on metabolomics. It is concluded that metabolomics could explain tramadol’s adverse reactions, including disruption of the GABA neurotransmitter system, phospholipid metabolism and energy metabolism, and may be a useful tool in identifying potential biomarkers following long-term tramadol abuse.

## Supplementary information

Supplementary file1 (DOCX 72 kb)

Supplementary file2 (DOCX 88 kb)

## Data Availability

The datasets supporting the conclusions of this article are included within the article.
