# Supplementary material for: Toxicology of tramadol following chronic exposure based on metabolomics of the cerebrum in mice
Source: Sci Rep. 2020 Jul 7;10:11130. doi: 10.1038/s41598-020-67974-8 (PMC7341866; doi:10.1038/s41598-020-67974-8)
Supplement: Supplementary file 1 — Supplementary file1 (DOCX 72 kb) [file 41598_2020_67974_MOESM1_ESM.docx]

Toxicology of tramadol following chronic exposure based on metabolomics of the cerebrum in mice

Wei Xia^1&^,Guojie Liu^2&^, Ziyi Shao^1^,Enyu Xu^1^, Huiya Yuan^1^, Junting Liu^1^, Lina Gao^1*^

(School of Forensic Medicine, China Medical University, Shenyang, 110014, China

School of Fundamental Sciences, China Medical University, Shenyang, 110014, China）
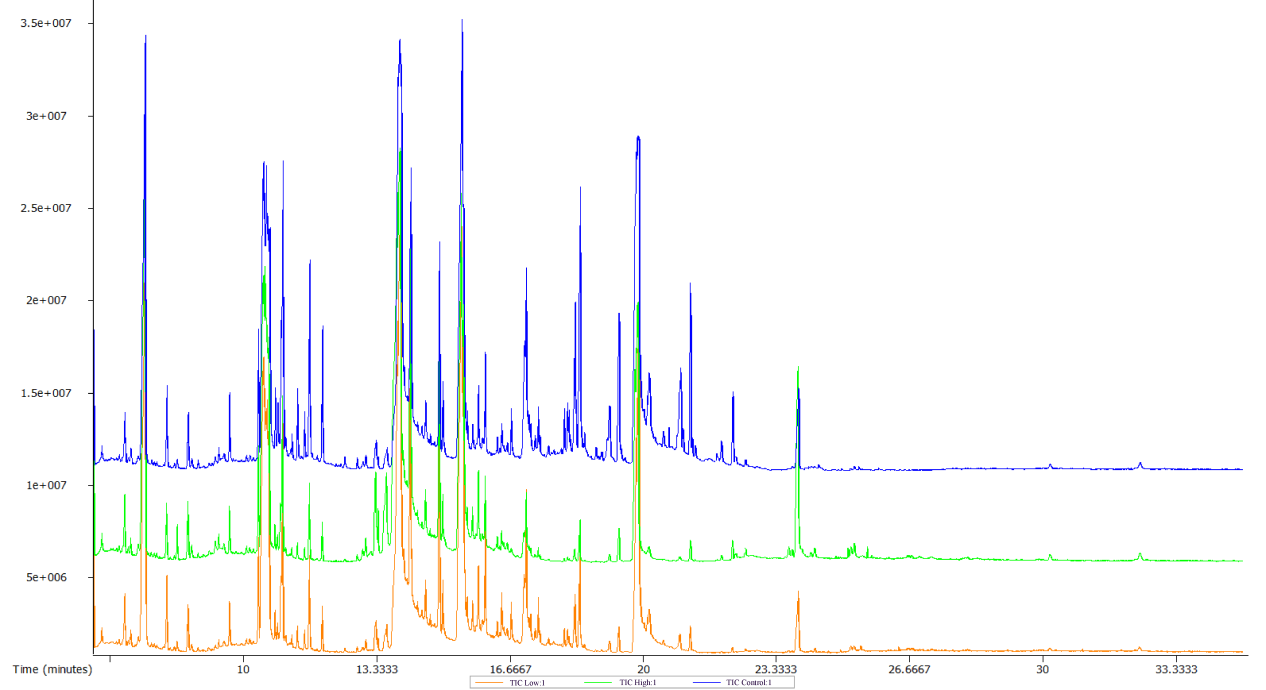


Supplementary Fig.1s Representative GC-MS spectra in cerebrum samples obtained from the control group and the tramadol group.
